# Type IX collagen deficiency enhances the binding of cartilage-specific antibodies and arthritis severity

**DOI:** 10.1186/ar1989

**Published:** 2006-07-03

**Authors:** Stefan Carlsen, Kutty Selva Nandakumar, Rikard Holmdahl

**Affiliations:** 1 Medical Inflammation Research, BMC I11, Lund University, SE-221 84 Lund, Sweden

## Abstract

Joint cartilage is attacked in both autoimmune inflammatory and osteoarthritic processes. Type IX collagen (CIX) is a protein of importance for cartilage integrity and stability. In this study we have backcrossed a transgenic disruption of the *col9a1 *gene, which leads to an absence of CIX, into two different inbred mouse strains, DBA/1 and B10.Q. None of the CIX-deficient mice developed observable clinical or microscopic osteoarthritis, but DBA/1 male mice had more pronounced enthesopathic arthritis, the so-called stress-induced arthritis. Both DBA/1 and B10.Q strains are susceptible to the induction of collagen-induced arthritis, and CIX deficiency in both strains led to the development of a more severe arthritis than in the controls. Induction of arthritis with monoclonal antibodies against type II collagen (CII) led to an earlier arthritis in the paws that also involved the knee joints. The antibodies used, which were specific for the J1 and the C1^I ^epitopes of CII, initiate their arthritogenic attack by binding to cartilage. The C1^I^-specific antibodies bound to cartilage better in CIX-deficient mice than in wild-type animals, demonstrating that the lack of CIX in cartilage leads to an increased accessibility of structures for antibody binding and thus making the joints more vulnerable to inflammatory attack. These findings accentuate the importance of cartilage stability; cartilage disrupted as a result of genetic disorders could be more accessible and vulnerable to an autoimmune attack by pathogenic antibodies.

## Introduction

Osteoarthritis (OA) and rheumatoid arthritis (RA) are both degenerative diseases of the joint and affect articular cartilage, synovium and bone with a loss of function and joint deformity. RA is, however, an inflammatory disease that involves a joint-specific autoimmune attack. Collagen type IX (CIX) is a protein associated with hyaline cartilage, together with collagen type II (CII) and XI (CXI). The triple helix of CIX is composed of three genetically distinct polypeptide-chains and belongs to the group of fibril-associated collagens with interrupted triple helices (FACIT). CIX molecules cover the surface of the heterotypic fibril of CII/CXI in a periodic fashion. Covalent cross-links between CII and CIX as well between other CIX molecules are formed [1-3], suggesting that CIX might form a macromolecular bridge between fibrils and with other matrix constituents, compromising the stiffness and tensile strength of cartilage [4]. Evidence for such a role comes from studies of transgenic mice with a truncated form [5] or complete deletion of the α1(IX) chain, leading to an absence of CIX in the cartilage [6]. Both types of mice have been reported to develop a mild form of OA in the knees. In addition, the importance of CIX was highlighted when a form of multiple epiphyseal dysplasia, EDM2, was linked to the *col9a2 *gene [7,8]. These patients developed irregular epiphyses with a gradual appearance of OA in the knees. Taken together, these observations suggest that CIX is important in maintaining the long-term stability of the articular cartilage.

The most widely used animal model for arthritis is collagen-induced arthritis (CIA), which has a disease course similar to that of RA, both in the spread of affected joints and in histopathological findings. CIA has a strong B-cell response, producing antibodies directed toward CII-specific structures [9,10], and these antibodies have been shown to be pathogenic in passive transfer experiments [11-15]. CII-specific T cells promoted the arthritis initiated by these antibodies [16-18]. A requirement for T cells in CIA was demonstrated by using anti-CD4 [19] or anti-TCRαβ [20] monoclonal antibodies and T-cell-deficient mice [21]. However, T cells alone cannot explain the pathology in CIA; hence both humoral immunity and cellular immunity were found to be absolutely essential [17]. Similarly, it is also possible to study arthritogenic pathways by using animal models other than CIA to pinpoint the effect of different disorders and thereby fine-tune the understanding of the underlying mechanisms of RA. Such models are collagen-antibody-induced arthritis (CAIA) and stress-induced arthritis (SIA).

In CAIA it is possible to study the inflammatory phase of the immune response without involving the primary phase by activating a cascade of reactions involving complement [22] and Fcγ receptors [23]. Neutrophils and macrophages are the major mediators of this inflammation [12]. CAIA is also capable of initiating arthritis independently of T and B cells, actually in the absence of either T or B cells, but not both enhanced the disease, arguing for a regulative role for these cells [16].

SIA is a spontaneous arthritis occurring in old male DBA/1 mice that are grouped from different litters. Macroscopically, it is similar to CIA, with edema and deformity of the hindpaws; however, SIA is primarily an enthesopathy characterized by the proliferation of fibroblasts and the formation of periarticular enthesophytes of cartilage and bone that can result in marginal ankylosis. In contrast to CIA, SIA is T-cell independent and shows minimal proliferative synovitis, without involving large cellular infiltrates [24].

Ankylosing enthesitis is a pathological feature in a group of chronic arthritides known as spondylarthropathies, with the same inflammatory mediators as in RA. IFN-γ (secreted probably by the innate immune system involving IFN-γ producing cells such as natural killer and myeloid cells) was shown to promote SIA [25]. It has been well documented that bone morphogenetic protein (BMP), a member of the transforming growth factor (TGF) superfamily, is involved in endochondral bone formation. Recently, an antagonist to BMP was shown to inhibit the onset and progression of SIA [26]; however, TGF-β1 injected directly into the knees induced enthesopathies [27]. The causative factor of spondylarthropathies is believed to be an endocrine disorder in humans [28] and might also be a compensatory mechanism of joint instability, supported by the stabilization process of osteoarthritic knees [29].

This study was designed to address whether the development of autoimmune arthritis as in RA might be dependent on cartilage quality. To address this question we used CIX mutant mice lacking the α1 (IX) chain [6] and assumed that this mouse might be more susceptible to inflammatory arthritis because of mild cartilage matrix destabilization. The data presented here confirmed our hypothesis that these mice developed a more severe arthritis in CIA and CAIA, which could be explained by an enhanced accessibility of CII-specific antibodies to cartilage. We also found that this mouse was more prone to developing enthesopathy but we could not reproduce earlier findings that the CIX deficiency leads to OA.

## Materials and methods

### Animals

**Figure 1:**
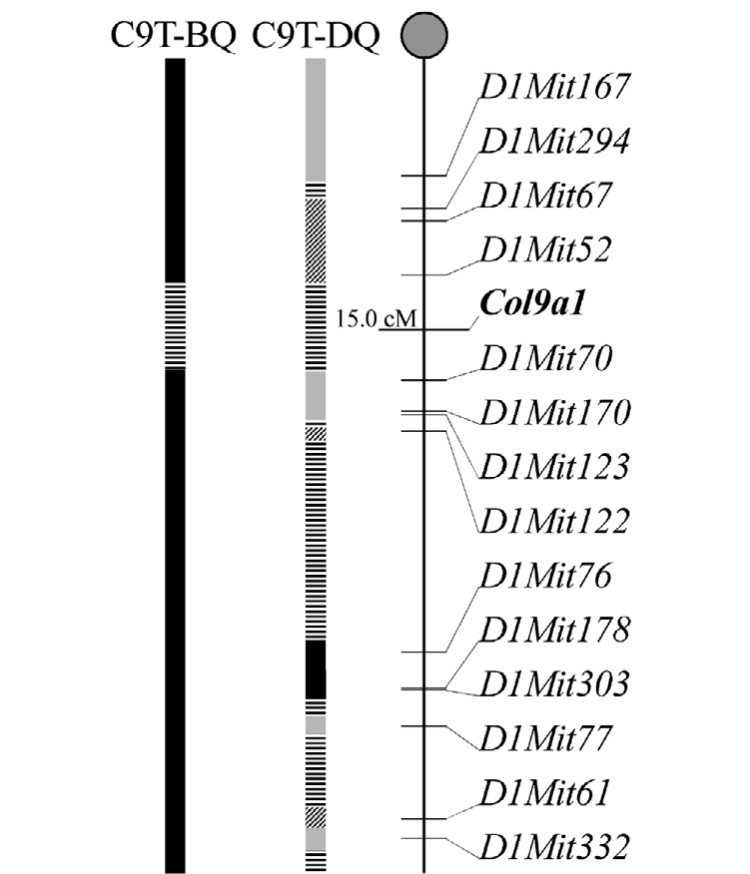
Genetic map of chromosome 1 around the *col9a1 *gene. Shown is a map of the genotyped chromosome 1 from 10th and 11th generations of mice backcrossed to B10.Q (C9T-BQ) and DBA/1 (C9T-DQ) strains, indicating the markers used and the position of *col9a1*. Chromosomal length and intermarker distances are proportional to the graphic length. The vertical bars specify background inheritance with C57bl mice (black bars), DBA/1 mice (grey bars) and 129 mice (hatched bars); linked markers and intermarker borders (striped bars) are also indicated.

The transgenic mice harboring mutant *col9a1 *have been described previously [6]; in brief, exon 8 of *col9a1 *was disrupted by inserting a phosphoglycerate kinase 1 (*pgk-1*) promoter-neomycin gene cassette. Homozygous mutant mice lacked both mRNA and polypeptide, but even though *col9a2 *and *col9a3 *transcription was normal the polypeptides were not detectable in cartilage. The CIX-deficient mice, denoted C9T, were then backcrossed to B10.Q/Rhd (originally from Jan Klein, Tübingen, Germany) and DBA/1/Rhd (originally from Jackson laboratories Inc., Bar Harbor, ME, USA) animals. Rhd indicates that these classical inbred mouse strains have been maintained in our laboratory for more than two decades. The two lines were further denoted by the suffixes -BQ and -DQ, to indicate B10.Q and DBA/1 backgrounds, respectively. The mice were backcrossed for 10 or 11 generations and the remaining fragment was analyzed with microsatellite markers between positions 6.5 and 43.1 centimorgans on chromosome 1 (see Figure 1 below). SinceC9T-BQ was determined as being free from genetic contamination, this line was kept by intercrossed breeding, and B10.Q animals were used as controls in the experiments.

All mice were kept in a climate-controlled environment with 12 hours light/12 hours dark cycles, housed in polystyrene cages containing wood shavings, standard rodent chow and water *ad libitum *in the Animal Department of Medical Inflammation Research in Lund, Sweden. All experiments were performed on age-matched mice, between 8 and 16 weeks of age. The Lund-Malmö laboratory animal ethical committee approved the animal experiments described in this article.

### Screening

Genomic DNA was prepared from the tip of tail or toe [30]. Transgenic mice were screened by PCR with primer pairs specific for the *NeoR *gene within the construct. Wild-type animals were determined by using another primer pair inside the *Col9a1 *gene that flanked the construct, yielding an incomplete product without amplification if the construct was present. Both primer pairs were run simultaneously for each sample during PCR, to yield on agarose gel electrophoresis two different products from each pair, depending on the haplotype.

### Induction and evaluation of arthritis

#### CIA

Mice were immunized intradermally at the base of the tail with 50 or 100 μg of CII, prepared from Swarm rat chondrosarcoma. All dosages were emulsified with complete Freund's adjuvant (Difco, Detroit, IL, USA) in a total volume of 100 μl. In early experiments, arthritis was followed by using the original, referred to as the simple, macroscopic score system for the four paws ranging from 1 to 3: 1 = swelling or redness in one joint or toe; 2 = more than one joint/toe or the ankle affected; 3 = severe arthritis in the entire paw. During later experiments the scoring protocol was improved and animals were scored by the system ranging from 0 to 15 per paw [31], referred to as the extended protocol. For comparison, scores used with the extended system have been converted from original documents for each individual and day in accordance with the following relation between extended and simple: 1 = 1, 2 to 10 = 2, and 11 to 15 = 3.

#### SIA

Spontaneous development of arthritis is influenced by hormonal and behavioral factors [32]. It has been stressed that the number of mice is critical (at least three), and grouping should be from sexually mature mice from different litters for the induction of SIA. Selection was used randomly to reduce cage variations. Arthritis was assessed as described above.

#### CAIA

To induce arthritis with anti-CII antibodies, GammaBind™ Plus Sepharose™ column (Amersham Biosciences, Uppsala, Sweden) purified IgG from the culture supernatants of B-cell hybridomas CIIC1 (C1^I ^epitope) and M2139 (J1 epitope) [33-35] were solubilized in PBS, sterile filtered and injected intravenously [12]. Each antibody (4.5 mg) was given as a cocktail on two occasions in a total volume of 400 μl. On day 5, 50 μg of lipopolysaccharide per mouse was injected intraperitoneally into all mice. Arthritis was assessed as described above.

### ELISA

ELISA was performed with sera obtained at day 35 from mice through retro-orbital plexus bleeding. Microtiter plates (Immunolon 2 HB; Thermo Labsystems, Franklin, MA, USA) were used, otherwise the protocol was followed as described elsewhere [36].

### Histology

CIIC1 (C1^I ^epitope, ARGLT), M2139 (J1 epitope, MPGERGAAGIAGPK), CIIC2 (D3 epitope, ARGAQGPPGATGFP), CIIF4 (F4 epitope, ERGLKGHRGFT) and CIIE8 (E8 epitope, LAGQRGIV) antibodies were biotinylated as described previously [33]; 100 μg of antibodies in 100 μl of PBS was then injected intraperitoneally into 1-day-old mice [37]. The mice were decapitated a day later and the hindlimbs were immediately embedded in OCT compound (Sakura Finetek, Zoeterwoude, The Netherlands), snap-frozen in isopentane and kept frozen at -70°C until cryosectioning. Staining was performed as described previously [38].

### Calculation of antibody binding

Sections were selected from the ankle region with homogenous, whole, round parts and from the same joint part as possible. All pictures were taken on the same occasion and stored as high-grade TIFF files, with SPOT advanced WIN v. 4.1 software (Digital Instruments Inc., Sterling Heights, MI, USA). All image analyses were performed at the same time with Easy Image Analysis 2000 v. 2.7.1.3 (Tekno Optik AB, Huddinge, Sweden) with threshold levels set as specifically as feasible and used unchanged throughout the analysis. The total area was marked manually and the software marked the stained area. The software calculated the stained area as a percentage of the total.

### Statistics

All ELISA data were first log-transformed and then used for calculations of the mean and an independent-samples *t *test, except for SD. Mean ELISA values are expressed as the antilog of transformed mean data (geo-mean). The Mann-Whitney *U *ranking test was used for score and one-way analysis of variance for onset. Pearson χ^2 ^with Yates's correction was calculated for incidence, or Fisher's exact test if groups were of less than five. In antibody-binding studies an independent-samples *t *test was used.

## Results

### Increased susceptibility to stress-induced arthritis but not osteoarthritis in CIX-deficient mice

To evaluate the susceptibility to arthritis in genetically pure inbred strains, the C9T mice were backcrossed into B10.Q and DBA/1 genetic backgrounds. These mouse strains express the MHC Aq class II gene, permitting susceptibility to CIA. In addition, DBA/1 males develop a spontaneous enthesopathy, denoted SIA, which readily develops after males are grouped together from different litters. The C9T mice were derived from a 129/sv line and were backcrossed for more than 10 generations followed by intercrossing and selection for minimal linked fragments, leaving a defined fragment around the *col9a1 *gene (Figure 1). C9T-BQ had a minimal 129-derived congenic fragment, whereas C9T-DQ had a larger congenic fragment that also contained DNA derived from both C57BL and 129 lines. The C57BL positive marker within C9T-DQ most probably originated from C57BL/6, which was used during transmission to a germline and then backcrossed with 129/sv to an inbred line [6]. Another embryonic stem 129 cell line, D3 [39], was used for electroporation but could not be distinguished from 129/sv by the microsatellite markers used. These strains seemed to have normal knee joints, because we did not find any microscopic or macroscopic sign of OA or other pathology in a large number of normal young and old (more than one year) mice of either strain. Samples were also taken from CIA and SIA experiments with littermate controls of both heterozygous and wild-type haplotypes, with additional inbred DBA/1 and B10.Q strains for negative comparison (data not shown).

To investigate whether the CIX-deficient mice were more prone to SIA, the males from different litters were grouped and followed for the development of arthritis. The disease typically started with swelling and erythema in the hind ankle and toes, which lasted for about ten weeks and then subsided leaving the joints deformed and stiff. Relapses were commonly seen after the deformity at periodic intervals. The CIX-deficient mice had a more severe disease than the wild-type or heterozygote controls (Table 1).

### Severity of collagen-induced arthritis is increased in CIX-deficient mice

**Figure 2:**
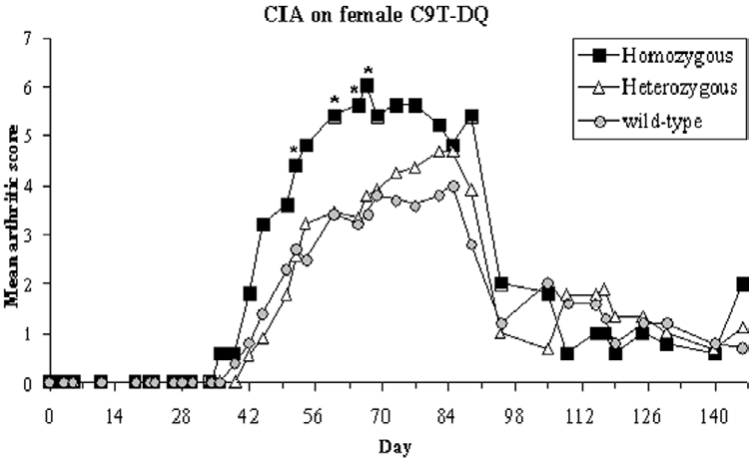
Severity of collagen-induced arthritis is increased in mice deficient in collagen type IX. The figure shows a comparison of arthritis development between littermate females of C9T-DQ from the intercrossed DBA/1 line, as in Table 1. Mean scores were calculated on arthritic animals. An asterisk indicates a significant difference (*p *< 0.05) between the homozygous group and the control groups (heterozygous and wild types).

To test whether the CIX deficiency makes the cartilage more susceptible to autoimmune-mediated inflammation, we immunized the mice with heterologous rat CII. DBA/1 mice are highly susceptible to CIA, and to avoid a possible influence of SIA we used only females. B10.Q mice are less susceptible to arthritis and hence only males were tested with a higher CII dosage. DBA/1 mice with CIX deficiency developed significantly more severe arthritis than the heterozygous or wild-type littermate controls (Table 1 and Figure 2). C9T-BQ animals also had significantly more severe arthritis than the wild type. The increase in severity was observed early after the onset and lasted until the arthritis in both the strains had subsided. The autoimmune response was not affected, because no difference in serum levels of antibodies against CII was observed in either C9T-DQ (Table 1) or in an early low-dosage experiment in C9T-BQ with 50 μg/ml CII (homozygous, 109.5 ± 129.2 μg/ml; heterozygous, 96.0 ± 98.7 μg/ml; wild type, 135.5 ± 230.4 μg/ml; means ± SD). These findings argue in favor of our hypothesis that CIX deficiency might regulate arthritis through greater exposure of the cartilage to inflammatory attack. To test this possibility we conducted passive transfer experiments with CII-specific arthritogenic antibodies.

### CIX-deficient B10.Q mice developed a more severe collagen-antibody-induced arthritis

**Figure 3:**
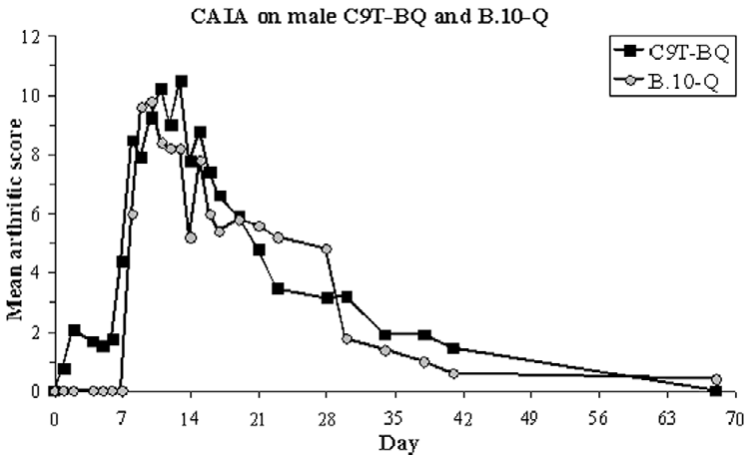
B10.Q mice deficient in collagen type IX developed a more severe collagen-antibody-induced arthritis. Arthritis development was followed in C9T-BQ and B10.Q mice injected with anti-collagen type II antibody, as in Table 1. Mean scores were calculated on arthritic animals.

To induce CAIA we used two well-defined monoclonal antibodies, CIIC1 and M2139. CIX-deficient B10.Q mice developed arthritis earlier (even before lipopolysaccharide injection) than the control group (Figure 3). After lipopolysaccharide injection, both groups developed arthritis with a peak at about day 10 and subsided at the same rate. Interestingly, some CIX-deficient mice had signs of arthritis in the knees (Table 1). This could be seen as gait disturbances as well as swelling and erythema of the knees. Comparable observations were observed in C9T-DQ mice, in which four out of nine mice developed knee inflammation after disease progression in the paws during onset and subsidence (data not shown).

### Anti-CII antibodies bind more efficiently to cartilage lacking CIX

**Figure 4:**
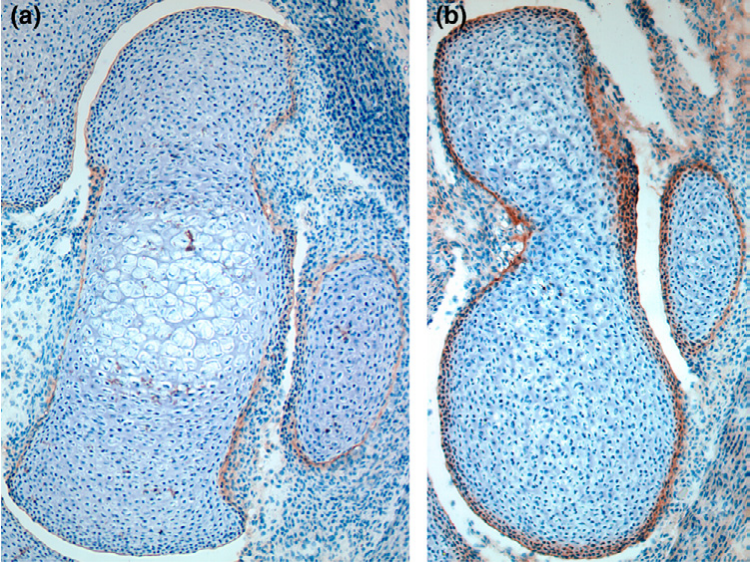
Improved binding of anti-collagen type II antibodies to cartilage deficient in collagen type IX. Examples of immunohistochemical staining of the ankle from the hind paws of newborn mice, injected intraperitoneally with 100 μg of biotinylated C1 antibodies, as described in the Materials and methods section. Pictures show the tibia to the far left and the talus and calcaneus to the far right. **(a) **Section from a B10.Q mouse; **(b) **the same area from a C9T-BQ mouse except that the tibia is out of the picture. In this example the calcaneus was used for calculations. The pictures were developed and handled identically.

It is possible that the deficiency of CIX might not only destabilize cartilage but could also affect the exposure of epitopes for antibody binding. To test this possibility, biotinylated monoclonal antibodies against the major B-cell epitopes of CII (C1, J1, C2, F4 and E8) were injected into newborn mice. In this screen there was an indication that the CIIC1 antibody could bind better to the cartilage from CIX-deficient mice than to that from controls. We extended this observation with a larger number of mice. We quantified the CIIC1 binding by developing a method in which both density and area could be measured objectively (Table 2 and Figure 4). Using this method, we showed that the CIIC1 monoclonal antibody had a denser staining of a larger area in CIX-deficient mice than in controls.

## Discussion

CIX-deficient mice have a subtle defect of the cartilage, which is here indicated by their higher susceptibility to developing spontaneous enthesopathic arthritis. Interestingly, these mice also developed a more severe autoimmune arthritis, as shown with the CIA model. Parts of this effect could be explained by the higher accessibility of CIX-deficient cartilage for binding of arthritogenic CII-specific antibodies.

CIX-deficient mice [6] have previously been shown to develop microscopic OA at one year of age, with loss of cartilage at the femoral and tibial joint surfaces and increased cartilage and bone formation at the periphery, leading to prominent shape changes. In transgenic mice with truncated CIX [5], OA was observed in mice up to one year old as a decreased intensity of Safranin O staining and roughening or erosion of the articular surface. In our study we could not detect either gait problems or histological OA. One reason for this might have been that we focused on mice with the CIX deficiency backcrossed to DBA/1 and B10.Q genetic backgrounds, which are not known to be sensitive for spontaneous OA, unlike the C57BL/6 and BALB/c [40] genetic backgrounds used in the previous studies. The development of spontaneous enthesopathy in DBA/1 mice is an indication of the presence of a good compensatory mechanism to preserve joint homeostasis [29], resulting in an improved repair mechanism for local injury [41]. However, enthesopathy in DBA/1 mice is a pathological event, with increased new bone formation in tissues where tendons and ligament attach, with abundant proliferative fibroblasts and chondrocytes. In our SIA experiments the homozygous mice developed a more severe disease, which suggests that the mediators released during stress affect chondrocytes profoundly, and chondrocytes might possibly try to compensate for the CIX deficiency by the overexpression of cartilage components.

To test our hypothesis about whether cartilage disorder could alter the course of arthritis as a result of enhanced accessibility to the immune system, we used our CIX-deficient mice in two different genetic backgrounds in CIA. In both these backgrounds a more severe development of arthritis was observed with CIX deficiency. This phenomenon could not be explained by increased turnover of the fragile matrix and, in turn, priming of autoreactive T cells or efficient tolerance induction, because the disease course was as self-limiting as in the controls. Similarly, there was no change in antibody titers, which argues against *de novo *priming of T cells. Still, there might have been a difference in the accessibility of cartilage to antibodies, because antibodies are shown to bind complement and to be involved in direct cell interactions via Fc receptors, which would certainly have an effect on disease progression. In fact, this was the case when tested with direct CII antibody injection: homozygous mutant mice developed arthritis much earlier than the controls and had more antibodies attached to the cartilage. This also ruled out the possibility of T-cells as the main promoter during the course of arthritic disease in this model, because antibody-mediated arthritis is T-cell independent.

Because CAIA experiments gave such a rapid response and also an apparent knee inflammation in both the mouse strains, we were prompted to perform *in vivo *binding studies with different monoclonal antibodies. Of the different antibodies used, CIIC1 binding to the C1^I ^epitope showed an increased binding to cartilage. The C1 epitope was identified as a major epitope in generating the antibody response to CII, and the various antibodies thus developed recognized different parts of the epitope: the C1^I ^epitope 359 to 363, the C1^II ^epitope 359 to 366 and the C1^III ^epitope 359 to 369 [35]. However, all antibodies are dependent on the first part of the epitope, where the CIIC1 antibody binds. The antibody response to the C1 epitope dominates the immune response in CIA in both mice [42] and rats [43].

The CIIC1 antibodies also impaired cartilage formation by cultured chondrocytes [44], strongly inhibited the self-assembly of CII *in vitro *[45] and caused disorganization of CII fibrils in the extracellular matrix without affecting chondrocyte morphology, along with increased matrix synthesis [46], and thus the antibodies directed to this epitope can contribute directly to cartilage destruction. Increased binding of CIIC1 to the CIX-deficient cartilage indicates that the C1^I ^epitope is more exposed for the CIIC1 antibody in the absence of CIX and more antibodies bind per CII molecule in the deeper layers of cartilage.

Interestingly, antibodies against the C1 epitope have also been shown to be associated with RA [47]. Clearly, the C1 epitope is not only important for its immunodominance but also contributes to matrix component interactions, signaling and stability. In contrast, murine antibodies reacting to the F4 epitope were not arthritogenic in CAIA and were associated with OA rather then RA [47]. Furthermore EDM1, another subgroup of multiple epiphyseal dysplasia, and pseudochondroplasia are linked to defects in cartilage oligomeric matrix protein (COMP) [48] and share pathogenesis with EDM2 [49], suggesting that COMP and CIX are interacting in the large polymeric network of cartilage. Deficiency of CIX might therefore also lead to instability and changed exposure of COMP. Increased serum levels of COMP have not been detected in different human OA, but it would be interesting to study COMP-mediated pathology in CIX-deficient mice.

Using CIX-deficient mice of two well-defined mouse inbred lines B10.Q and DBA/1, which are susceptible to CIA, we have tested the possibility that disordered cartilage alters susceptibility to autoimmune arthritis. We found that the lack of CIX increased the binding of antibodies to the major epitopes on CII in cartilage and led to a higher susceptibility to both CIA and CAIA. RA probably represents a wide variety of diseases with different initiation events. It could in fact include subgroups that have a mild cartilage defect genetically. Such groups could be dormant but the presence of fragile cartilage could expose B-cell epitopes to binding of autoreactive antibodies. The bound antibodies could then initiates an inflammation with the release of cartilage antigens, thereby further driving an autoimmune attack towards cartilage proteins. This study supports the idea that a mild disruption to cartilage increases the severity of arthritis.

## Conclusion

To understand the importance of CIX for cartilage integrity and stability and in the disease process of arthritis, we used a transgenic disruption of the *col9a1 *gene, leading to an absence of CIX, in two different genetic backgrounds (DBA/1 and B10.Q). None of the CIX-deficient mice developed OA, but the control DBA/1 male mice had more pronounced enthesopathic arthritis. Both DBA/1 and B10.Q strains are susceptible to the induction of CIA, and CIX deficiency in both strains led to the development of a more severe arthritis than in the controls. Induction of arthritis with monoclonal antibodies against CII led to earlier arthritis in the paws and also involved the knee joints. The C1^I^-specific antibodies bound to cartilage better in CIX-deficient mice than in wild type animals. This finding demonstrates that a lack of CIX in cartilage leads to an increased accessibility of structures for antibody binding and thus makes the joints more vulnerable to an inflammatory attack. These observations accentuate the importance of cartilage stability; disrupted cartilage due to genetic disorders might be more accessible and vulnerable to autoimmune attack by pathogenic antibodies.

## Abbreviations

CAIA = collagen-antibody-induced arthritis; CII = type II collagen; CIX = type IX collagen; CIA = collagen-induced arthritis; COMP = cartilage oligomeric matrix protein; ELISA = enzyme-linked immunosorbent assay; IFN = interferon; OA = osteoarthritis; PBS = phosphate-buffered saline; PCR = polymerase chain reaction; RA = rheumatoid arthritis; SIA = stress-induced arthritis.

## Competing interests

The authors declare that they have no competing interests.

## Authors' contributions

SC and KSN performed the experiments under the supervision of RH. All authors read and approved the final manuscript.

## Figures and Tables

**Table 1:** Summary of experiments conducted on C9T mice

			Arthritic/total		Simple maximal	Extended maximal	Anti-CII antibody
Experiment	Strain	Group	(*n*/*n*)	Day of onset	arthritic score	arthritic score	titer (μg/ml)
CIA^a^	C9T-DQ	Homozygous	5/11	60.6 ± 39.1	8.6 ± 2.7^e^	nd	371.7 ± 359.3
CIA^a^	C9T-DQ	Heterozygous	9/17	50.9 ± 8.2	5.4 ± 2.0^e^	nd	532.1 ± 529.2
CIA^a^	C9T-DQ	Wild type	10/17	55.6 ± 19.0	5.1 ± 2.9^e^	nd	454.3 ± 489.0
CIA^b^	C9T-BQ	Homozygous	6/9	62.8 ± 13.6	8.3 ± 2.0	29.9 ± 15.3^e^	nd
CIA^b^	B10.Q	Wild type	5/9	66.4 ± 17.3	6.0 ± 2.3	23.8 ± 8.2^e^	nd
SIA^c^	C9T-DQ	Homozygous	9/18	44.3 ± 18.4	3.1 ± 1.2^f^	nd	nd
SIA^c^	C9T-DQ	Heterozygous	19/26	43.7 ± 30.1	2.7 ± 1.6^f^	nd	nd
SIA^c^	C9T-DQ	Wild type	12/17	39.2 ± 23.8	2.0 ± 0.9^f^	nd	nd
CAIA^d^	C9T-BQ	Homozygous Ab	13/20	5.5 ± 3.0^g^	4.8 ± 2.8	12.8 ± 11.2	nd
CAIA^d^	C9T-BQ	Homozygous PBS	0/8	-	-	-	nd
CAIA^d^	B10.Q	Wild-type Ab	5/8	8.4 ± 0.5^g^	5.6 ± 3.0	11.2 ± 8.5	nd

Where errors are shown, results are means ± SD. ^a^One generation intercrossed C9T-DQ and littermate control females were immunized with 50 μg of CII. ^b^Pure lines of C9T-BQ and B10.Q male mice were immunized with 100 μg of CII. ^c^One generation intercrossed male C9T-DQ and littermate controls were randomly and pooled blind. ^d^Anti-CII antibody-induced arthritis on pure line of male C9T-BQ (homozygous Ab) and male B10.Q (wild-type Ab), and controls injected with PBS (homozygous PBS). ^e^Homozygous mice were significantly higher in score (*p *< 0.05) than both heterozygous (if present) and wild type, respectively. There were no other significant differences between the groups within the experiments. ^f^Homozygous mice were significantly higher in score (*p *= 0.025) than wild type but not heterozygous. There were no other significant differences between the groups. ^g^Homozygous mice that received Ab had a significantly earlier onset (*p *= 0.035) with six arthritic mice before lipopolysaccharide injection, than their wild-type counterpart. Two homozygous mice injected with Ab also developed knee inflammation at days 4 and 6, which were not seen in wild-type Ab. CAIA, collagen-antibody-induced arthritis; CIA, collagen-induced arthritis; CII, type II collagen; nd, not determined; SIA = stress-induced arthritis.

**Table 2:** Anti-type II collagen antibody staining on neonatal cartilage

Strain	*n*	Area fraction^a^
C9T-BQ	8	17.2 ± 9.8^b^
B10.Q	8	6.5 ± 1.8^b^

Sections were obtained and stained as described in materials and methods from pure-line homozygous C9T-BQ and wild-type controls (B10.Q). Where errors are shown, results are means ± SD. ^a^Area fraction is calculated as the stained area as a percentage of the total area, from one joint per individual. ^b^C9T-BQ animals had a significantly more densely stained area (*p *= 0.009) than their wild-type counterpart.
